# PPARδ Agonist GW501516 Suppresses the TGF-β-Induced Profibrotic Response of Human Bronchial Fibroblasts from Asthmatic Patients

**DOI:** 10.3390/ijms24097721

**Published:** 2023-04-23

**Authors:** Milena Paw, Dawid Wnuk, Zbigniew Madeja, Marta Michalik

**Affiliations:** Department of Cell Biology, Faculty of Biochemistry, Biophysics and Biotechnology, Jagiellonian University, Gronostajowa 7, 30-387 Kraków, Poland

**Keywords:** transforming growth factor-β, human bronchial fibroblasts, myofibroblast transitions, asthma, PPARδ, GW501516, GSK0660

## Abstract

The airway wall remodeling observed in asthma is associated with subepithelial fibrosis and enhanced activation of human bronchial fibroblasts (HBFs) in the fibroblast to myofibroblast transition (FMT), induced mainly by transforming growth factor-β (TGF-β). The relationships between asthma severity, obesity, and hyperlipidemia suggest the involvement of peroxisome proliferator-activated receptors (PPARs) in the remodeling of asthmatic bronchi. In this study, we investigated the effect of PPARδ ligands (GW501516 as an agonist, and GSK0660 as an antagonist) on the FMT potential of HBFs derived from asthmatic patients cultured in vitro. This report shows, for the first time, the inhibitory effect of a PPARδ agonist on the number of myofibroblasts and the expression of myofibroblast-related markers—α-smooth muscle actin, collagen 1, tenascin C, and connexin 43—in asthma-related TGF-β-treated HBF populations. We suggest that actin cytoskeleton reorganization and Smad2 transcriptional activity altered by GW501516 lead to the attenuation of the FMT in HBF populations derived from asthmatics. In conclusion, our data demonstrate that a PPARδ agonist stimulates antifibrotic effects in an in vitro model of bronchial subepithelial fibrosis. This suggests its potential role in the development of a possible novel therapeutic approach for the treatment of subepithelial fibrosis during asthma.

## 1. Introduction

The constantly increasing prevalence of asthma [1,2,3,4] and the lack of effective agents that target the reversal of functional impairment of asthmatic bronchi remain the main challenges of contemporary medicine [5]. In asthma treatment, drugs extinguishing the chronic inflammation and bronchodilators allow for controlling asthma but have a negligible effect on the structural changes in the bronchi [5]. Recent studies indicate that inflammation and remodeling of asthmatic bronchi can be driven independently during asthma [6,7,8]. Therefore, the ideal drug or treatment strategy should target both processes.

Airway remodeling observed in asthmatic patients is a complex process including epithelial disintegrity and functional impairment of its interactions with deeper parts of the bronchi (especially fibroblasts) within the epithelial–mesenchymal trophic unit [9,10]. It leads to the progression of subepithelial fibrosis through the uncontrolled activation of locally resident fibroblasts into highly contractile myofibroblasts (fibroblast to myofibroblast transition; FMT). Under chronic exposure to pro-inflammatory cytokines, mainly transforming growth factor-β (TGF-β), these cells acquire features characteristic of smooth muscle cells, such as a significant enhancement of α-smooth muscle actin (α-SMA) incorporated into stress fibers. On the other hand, myofibroblasts are characterized by significantly enhanced secretory activity for extracellular matrix (ECM) proteins, such as collagens, fibronectin, and tenascin C [11]. It should be highlighted that myofibroblasts play a role in maintaining tissue homeostasis and wound healing, but they sometimes escape from the tissue regime and remain insensitive to pro-apoptotic signals [11,12,13]. This phenomenon is observed in different pathological states, including asthma, where an increased number of myofibroblasts are observed in most bronchial tissue specimens derived from an asthmatic patient, causing subepithelial fibrosis [14].

Numerous reports indicate that the dysfunction of glucose and lipid metabolism is one of the major risk factors for asthma progression [15,16,17,18]. The dysregulated lipid status in obesity affects asthma manifestations, and standard therapy returns poor results in obese patients [19]. Therefore, new additional therapeutic strategies and drugs are urgently needed for patients with obesity-related asthma [16,20]. For this reason, the effects of clinically used anti-hyperlipidemic drugs on asthma progression are intensively studied [21,22,23]. Fenofibrate, known as a lipid-lowering agent and a peroxisome proliferator-activated receptor-alpha (PPARα) agonist, decreases experimental pulmonary fibrosis (PF) and subepithelial fibrosis in asthma [23,24]. PPARs are part of the hormone receptor superfamily, including PPARα, PPARδ (also called β), and PPARγ, activated by subtype-specific or pan-PPAR agonist ligands that promote their nuclear translocation and regulation of gene transcription by binding to their peroxisome proliferator response elements (PPREs) [25]. The involvement of nuclear PPARs in the attenuation of cardiac, dermal, and lung fibrosis has previously been reported [26,27,28,29], but its contribution to subepithelial fibrosis and TGF-β-induced FMT associated with asthma remains poorly understood. In particular, the physiological functions of PPARδ remain unknown and unexplained.

Based on the literature data, two compounds were selected for this study: GW501516, a strong PPARδ agonist, and the PPARδ antagonist GSK0660. The former has shown a high safety profile in various in vitro and in vivo models [30,31,32,33,34,35], and is also known as a cardarine—a supplement for athletes supporting muscle mass building and body fat reduction. GSK0660 has often been used in combination with GW501516 in different experimental approaches [32,33,34,35,36]. GSK0660 is a potent selective PPARδ antagonist that also has inverse agonist activity when used alone [36]. It was documented that in a standard cell-based Gal4 chimera/reporter antagonist assay, GSK0660 antagonized 100% of the activity of PPARδ with a pIC50 of 6.53 (above 10 μM), and was inactive on PPARα and PPARγ [36]. The high PPARδ antagonistic potential of GSK0660 corresponds to its well-documented anti-inflammatory effect [37,38], and a lack of bioavailability upon systemic administration [36]. Therefore, in this study, we investigated the effect of the PPARδ agonist GW501516 and the PPARδ antagonist GSK0660 administered alone or in combination on the phenotypic plasticity of HBF populations during TGF-β-induced subepithelial fibrosis in asthma.

## 2. Results

### 2.1. PPARδ Agonist GW501516 and PPARδ Antagonist GSK0660 Administered Separately or in Combination Inhibit the Proliferation and Viability of HBFs in a Dose-Dependent Manner

**Figure 1:**
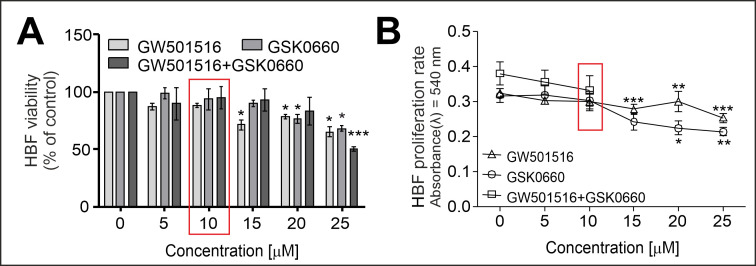
The PPARδ agonist GW501516 and antagonist GSK0660 administered alone or in combination to HBF cultures had a negligible effect on cell viability and proliferation. HBF populations (*n* = 7, all in duplicate) were exposed to increasing concentrations (0–25 µM) of GW501516 and GSK0660 administered alone or in combination (0–10 µM) for four days. (**A**) The viability of HBFs was determined using an FDA+/EtBr- assay. (**B**) The proliferation rates were determined using a crystal violet assay, and the absorbances were measured spectrophotometrically (λ = 540 nm). The results are expressed as a percentage of the control and presented as the mean ± SEM. Statistical significance was tested using the nonparametric Kruskal–Wallis test with Dunn’s multiple comparisons post hoc test; * *p* < 0.05, ** *p* < 0.01, *** *p* < 0.001. The red box indicates the concentration used for further analyses (10 µM).

To select the optimal concentration of the tested compounds, we cultured HBFs in a serum-free medium containing increasing concentrations of GW501516 and GSK0660 administered separately or in combination for four days. To verify the effect of the tested ligands on the viability of HBFs, we performed an FDA+/EtBr- assay. This test revealed a lack of a significant cytotoxic effect of the tested compounds on the HBFs at concentrations up to 10 µM (Figure 1A). We did not observe any anti-proliferative effects of the tested compounds at concentrations up to 10 µM (Figure 1B). Based on these results, we used the 10 µM concentration of the tested ligands for further experiments.

### 2.2. The TGF-β-Induced Myofibroblastic Transitions of HBFs Are Suppressed after PPARδ Agonist Administration

**Figure 2:**
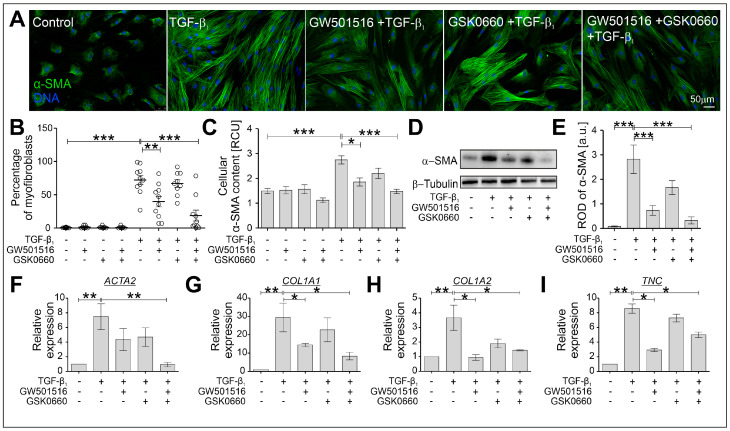
The profibrotic response of TGF-β_1_-stimulated HBFs was significantly attenuated by GW501516 when administered alone or in combination with GSK0660. HBFs derived from asthmatic patients were cultured in a serum-free medium containing GW501516 and GSK0660 alone or in combination, and in the absence or presence of TGF-β_1_ (5 ng/mL) for four days. (**A**) The FMT potential of HBFs was determined via immunostaining for α-SMA (green) with DNA (blue) visualization. Representative images are presented. Scale bar = 50 µm. (**B**) The percentage of myofibroblasts in HBF cultures (*n* = 10) are presented in the graph. The α-SMA content in the HBFs was determined via (**C**) in-cell ELISA (*n* = 8; each condition in triplicate) and (**D**,**E**) Western blotting (*n* = 3). RCU–relative colorimetric units. The relative expressions of selected FMT-related genes: (**F**) *ACTA2* (α-smooth muscle actin), (**G**) *COL1A1*, (**H**) *COL1A2* (collagens 1a1 and 1a2), and (**I**) *TNC* (tenascin C), in HBFs (*n* = 5) cultured for 24 h under the conditions described above were determined via real-time PCR. The results are presented as 2^−ΔΔCt^ mean values in relation to the control gene (*GAPDH*). The results are presented as the mean ± SEM. Statistical significance was tested using the nonparametric Kruskal–Wallis test with Dunn’s multiple comparisons post hoc test; * *p* < 0.05, ** *p* < 0.01, *** *p* < 0.001.

Further analyses were performed to estimate the effect of the PPARδ agonist and antagonist administered alone or in combination on the TGF-β-induced FMT in HBF populations (*n* = 10) after four days of culture. It has been frequently described that the phenotypic shifts of HBFs derived from asthmatic patients are effectively induced by TGF-β_1_ [10,22,23,39,40,41,42]. The strong stimulatory effect of TGF-β_1_ on the FMT potential of HBFs was significantly attenuated by GW501516 (Figure 2A,B). The observed effect was confirmed in the analyses of the α-SMA content in HBFs at the protein (Figure 2C–E) and gene expression levels (*ACTA2*; Figure 2F). A similar trend was observed in the analyses of other gene expressions of myofibroblast-related markers such as collagen 1a1 (encoded by *COL1A1*), collagen 1a2 (encoded by *COL1A2*), and tenascin C (encoded by *TNC*), where the strong stimulatory effect of TGF-β_1_ on the expression of these genes was downregulated in GW501516-treated HBFs (Figure 2G–I). A slight inhibitory effect of the PPARδ antagonist GSK0660 on the FMT potential and the expression of α-SMA as well as other myofibroblast-related markers was observed in TGF-β_1_-stimulated HBF populations. Moreover, administering the PPARδ agonist and PPARδ antagonist simultaneously led to a much stronger antifibrotic response of TGF-β_1_-treated HBFs, in which the expression of all the myofibroblast-related markers tested at the gene (Figure 2F–I) and protein levels (Figure 2C–E) was significantly decreased. The FMT potential (Figure 2B) was also significantly attenuated. These observations demonstrate the relatively strong inhibitory effect of the PPARδ agonist on the TGF-β_1_-induced myofibroblastic transitions of HBFs.

**Figure 3:**
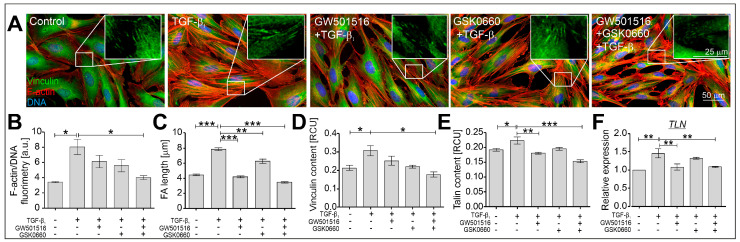
The actin cytoskeleton architecture in TGF-β-treated HBFs was modulated by GW501516 alone or in combination with GSK0660. HBFs derived from asthmatic patients (*n* = 3) were cultured in a serum-free medium containing GW501516 and GSK0660 alone or in combination, and in the absence or presence of TGF-β_1_ (5 ng/mL) for 24 h. (**A**) Representative images of HBFs immunostained for vinculin (green) and F-actin (red) with visualization of DNA (blue) are presented; scale bar = 50 µm. Vinculin-rich focal adhesion sites are shown enlarged in boxes; scale bar = 25 µm. (**B**) F-actin fluorimetry was quantified in relation to DNA fluorescence and is presented in the graph (*n* = 3, 260 cells/condition). (**C**) The lengths of vinculin-rich focal adhesion sites were measured and are presented in the graph. (**D**,**E**) The contents of focal-adhesion-related proteins vinculin and talin, were determined via in-cell ELISA (*n* = 8; each condition in triplicate). (**F**) The relative expression of *TLN* (talin) in HBFs (*n* = 3) cultured for 24 h under the conditions described above was determined using real-time PCR. The results are presented as 2^−ΔΔCt^ mean values in relation to the control gene (*GAPDH*). RCU–relative colorimetric units. The results are presented as the mean ± SEM. Statistical significance was tested using the nonparametric Kruskal–Wallis test with Dunn’s multiple comparisons post hoc test; * *p* < 0.05, ** *p* < 0.01, *** *p* < 0.001.

Upregulation of α-SMA in HBFs derived from asthmatic patients exposed to TGF-β is usually associated with reorganization of the actin cytoskeleton and focal adhesions (FAs), and this has been described previously [23,40,43]. For this reason, we checked the effect of the PPARδ agonist and antagonist administered alone or in combination on the actin cytoskeleton architecture in TGF-β_1_-treated HBF populations (*n* = 3) after 24 h of culture. Immunofluorescent studies revealed that the thickened microfilaments observed in TGF-β_1_-exposed HBFs were thinner in the cells treated with GW501516 and GSK0660 administered alone or in combination (Figure 3A). The quantification of the F-actin fluorescence signal in relation to DNA fluorescence confirmed the effect of the tested compounds on the architecture of the actin cytoskeleton in the HBFs. In particular, we observed a decrease in the fluorescence signal of F-actin in TGF-β_1_-treated HBFs in the presence of GW501516 and GSK0660 administered in combination (Figure 3B). Furthermore, the significantly greater number of vinculin-rich focal adhesion sites observed in TGF-β_1_-stimulated HBFs (Figure 3A, inserts) and quantified (Figure 3C) were strongly reduced in the cells treated with GW501516 alone or in combination with GSK0660. The effect of the PPARδ antagonist on the length of vinculin-rich Fas was weaker than in the cells treated with the PPARδ agonist. This observation was confirmed by immunoenzymatic analyses of the vinculin content in HBFs treated with TGF-β_1_ in the presence of GW501516 administered in combination with GSK0660 (Figure 3D). Analyses of other FA-related protein contents in TGF-β_1_-stimulated HBFs also revealed the inhibitory effect of GW501516 administered alone or in combination with GSK0660 on the level of talin at the protein (Figure 3E) and gene expression levels (Figure 3F). These observations indicate the inhibitory effect of GW501516 on the actin cytoskeleton architecture in TGF-β_1_-treated HBFs.

### 2.3. The TGF-β-Induced Upregulation of Cx43 Is Strongly Suppressed in HBFs Treated with GW501516 and GSK0660 Alone or in Combination via Smad-Dependent Signaling

**Figure 4:**
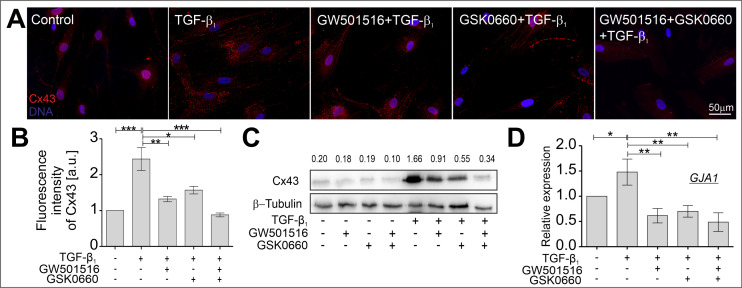
GW501516 and GSK0660, when administered alone or in combination, suppressed the TGF-β_1_-induced upregulation of Cx43 in HBF populations. HBFs derived from asthmatic patients were cultured in a serum-free medium containing GW501516 and GSK0660 alone or in combination, and in the absence or presence of TGF-β_1_ (5 ng/mL) for four days. (**A**) Representative images of HBFs immunostained for Cx43 (red) with DNA visualization (blue) are presented. Scale bar = 50 µm. Cx43 levels in HBFs were determined via (**B**) the quantification of the fluorescence signal in the collected images (*n* = 5) or via (**C**) Western blot analyses (*n* = 1, densitometry is presented above the bands). (**D**) The relative expression of *GJA1* (the Cx43-encoding gene) in HBFs (*n* = 3) cultured for 24 h under the conditions described above was determined using real-time PCR. The results are presented as 2^−ΔΔCt^ mean values in relation to the control gene (*GAPDH*). All results are presented as the mean ± SEM. Statistical significance was tested using the nonparametric Kruskal–Wallis test with Dunn’s multiple comparisons post hoc test; * *p* < 0.05, ** *p* < 0.01, *** *p* < 0.001.

It was previously reported that the transmembrane protein connexin 43 (Cx43) plays a crucial role in the induction of phenotypic shifts in TGF-β_1_-treated HBFs derived from asthmatic patients via the regulation of Smad-dependent signaling, and the incorporation of α-SMA into microfilaments [23,39]. In this study, the effect of the compounds tested, a PPARδ agonist and a PPARδ antagonist administered alone or in combination, on the levels of Cx43 in TGF-β_1_-stimulated HBFs was investigated. Using immunofluorescence analyses, the content and intracellular localization of Cx43 were visualized in HBFs cultured in the absence or presence of TGF-β_1_ with or without the PPARδ agonist and PPARδ antagonist administered alone or in combination (Figure 4A). The upregulation of Cx43 in HBFs treated with TGF-β_1_ was significantly decreased in cells cultured in the presence of the tested compounds. To confirm these observations, the quantification of the Cx43 fluorescence signal was performed in relation to DNA fluorescence (Figure 4B), and the analysis of the Cx43 protein (Figure 4C) and transcript (Figure 4D) levels. The results indicate the inhibitory effect of GW501516 and GSK0660 alone or in combination on the Cx43 protein and transcript levels in TGF-β1-treated HBFs.

**Figure 5:**
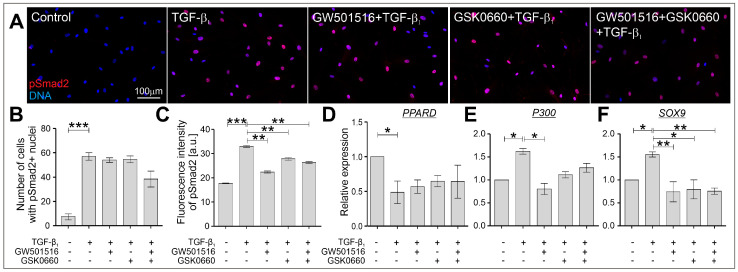
Canonical TGF-β_1_/Smad2-dependent signaling was modulated in HBFs in response to the administration of GW501516 and GSK0660 alone or in combination. HBFs derived from asthmatic patients were cultured in a serum-free medium containing GW501516 and GSK0660 alone or in combination, and in the absence or presence of TGF-β_1_ (5 ng/mL) for 1 h. (**A**) Representative images of HBFs immunostained for pSmad2 (red) with DNA visualization (blue) are presented. Scale bar = 100 µm. (**B**) The number of cells expressing pSmad2 in the nuclei area was quantified. (**C**) The levels of pSmad2 in the nuclei area of HBFs were determined via the quantification of the fluorescence signal in the collected images (*n* = 4; 420 cells/condition). The relative expression of genes encoding (**D**) PPARδ (*PPARD*), (**E**) the p300 cofactor (*P300*), and (**F**) the Sox9 transcription factor (*SOX9*) were measured using real-time PCR. The results are presented as 2^−ΔΔCt^ mean values in relation to the control gene (*GAPDH*). The results are presented as the mean ± SEM. Statistical significance was tested using the nonparametric Kruskal–Wallis test with Dunn’s multiple comparisons post hoc test; * *p* < 0.05, ** *p* < 0.01, *** *p* < 0.001.

Enhanced activation of Smad2/3-mediated signaling in the TGF-β-treated HBFs from asthmatic patients was observed and described previously, which led to the upregulation of several profibrotic genes, including *ACTA2*, *COL1A1*, and *COL1A2* [23,39,41,44,45]. To verify the effect of the tested compounds, a PPARδ agonist and a PPARδ antagonist administered alone or in combination, on the intracellular localization and phosphorylation levels of the Smad2 protein in TGF-β_1_-stimulated HBFs, we used immunofluorescent analyses. According to our previous reports [40,41], TGF-β_1_ strongly induced nuclear translocation and upregulation of the Smad2 phosphorylation level (Figure 5A–C). Although GW501516 and GSK0660 administered alone or in combination to TGF-β_1_-treated HBFs AS did not affect the number of cells with pSmad2-positive nuclei (Figure 5B), the quantification of the fluorescence signal of pSmad2 in relation to DNA fluorescence revealed significant attenuation of its phosphorylation and activation. Furthermore, the expression level of the ligand-activated transcription factor PPARδ downregulated by TGF-β_1_ was only slightly increased in response to the administration of the tested compounds (Figure 5D). Due to the fact that the transcriptional activity of pSmad2 is regulated by p300 acetyltransferase and Sox9 [46,47], we checked the expression levels of *P300* (Figure 5E) and *SOX9* (Figure 5F). The upregulation of *P300* and *SOX9* in the TGF-β_1_-treated HBFs was significantly attenuated by the PPARδ agonist and PPARδ antagonist administered alone or in combination. These results suggest that GW501516 and GSK0660 administered alone or in combination attenuate the profibrotic response of TGF-β_1_-treated HBFs via cooperative signaling dependent on the Smad2/p300/Sox9 transcriptional activity.

## 3. Discussion

The progression of subepithelial fibrosis in asthmatic bronchi is associated with the uncontrolled and constitutive activity of myofibroblasts that are responsible for the thickening of the airway wall, with a concomitant decrease in the lumen of the bronchi [48]. Unfortunately, the structural and functional impairments of the bronchi remain irreversible at this time due to the lack of effective antifibrotic therapies dedicated to asthmatic patients [5]. In vitro cultures of human bronchial fibroblasts established from bronchoscopy biopsies derived from asthmatic patients are a useful tool for conducting research on the mechanisms of FMT and subepithelial fibrosis. This has been well documented in our previous reports [10,22,23,39,40,41,42]. Fully controlled conditions of HBF cultures in vitro can be modulated by the addition of exogenous TGF-β to mimic the microenvironmental conditions observed in the bronchi of asthmatics. Using this in vitro model, we previously reported that the FMT potential of HBFs from asthmatics is significantly enhanced compared to their non-asthmatic counterparts, which is associated with the disturbed balance between the activity of profibrotic Smad2/3-mediated signaling and antifibrotic Smad1/5/9-mediated signaling [39,40,41,42,44]. The regulation and activation of profibrotic Smad2/3-dependent signaling leading to the enhanced transcription of profibrotic genes in HBFs are associated with the functions and levels of Cx43, a transmembrane protein that coordinates intercellular communication through gap junctions crucial for maintaining tissue homeostasis [23,39]. Populations of HBFs derived from asthmatic patients cultured in vitro also enable researchers to track and investigate the function of other proteins or transcription factors (e.g., p38) during FMT [40]. Furthermore, this model is convenient for testing the effects of different approved drugs for the treatment of other diseases on the FMT efficiency of HBFs from asthmatics, as well as the mechanisms of their action. As previously reported, lovastatin and fenofibrate, which have been routinely used in the clinical treatment of hyperlipidemia patients, showed a protective effect on the TGF-β-induced activation of HBFs from asthmatics [22,23].

Our focus on the effect of anti-hyperlipidemic drugs in the context of asthma progression was the result of several reports indicating that asthma progression with enhanced remodeling of the airways is associated with metabolic dysfunctions, such as obesity or hyperlipidemia, and dysregulation of the function of PPARs [18,49]. Fenofibrate is known as an agonist of PPARα, and its inhibitory effect on bronchial [23], lung [24,50], subretinal [51], and liver [52] fibrosis has been observed. However, little is known about the involvement of another type of peroxisome proliferator-activated receptor, PPARδ, in fibrosis-related processes, especially in asthma subepithelial fibrosis.

Several studies presented the anti-inflammatory effect of PPARδ activation by high-affinity ligands such as GW501516 and GW0742 in murine models of lipopolysaccharide (LPS)-mediated pulmonary inflammation [30,31]. In those studies, the concentrations of pro-inflammatory cytokines such as IL-6, IL-1β, TNFα, and granulocyte macrophage-colony stimulating factor (GM-CSF) in bronchial alveolar lavage fluid (BALF) and leukocyte recruitment into the lung tissue were significantly decreased. However, it has been shown that in the murine model of ovalbumin-induced asthma, GW501516 did not affect the airway inflammation measured by allergen-induced bronchoalveolar lavage eosinophil and lymphocyte influx [53]. In another study, GW0742 inhibited the proliferation of mouse or human lung fibroblasts [27]. Our results indicated a lack of a significant inhibitory effect of GW501516 at a concentration of 10 µM on the proliferation rate of HBFs and their viability after 4 days of culture. However, the lack of any data on the impact of PPARδ agonists on the myofibroblastic transition observed in the airways of asthmatic patients led us to investigate this phenomenon. Thus, this study fills this gap and remains the first report on the effect of the PPARδ agonist GW501516 on the FMT efficiency in populations of HBFs derived from asthmatic patients cultured in vitro.

In this study, we showed that the PPARδ agonist GW501516 significantly attenuated the TGF-β_1_-induced FMT potential in HBFs derived from asthmatic patients, measured by the percentage of myofibroblasts in the tested HBF populations. In our model, it was associated with decreased levels of myofibroblast-related genes (*ACTA2*, *COL1*, and *TNC*) and proteins, such as α-SMA. Similar observations have previously been described in populations of human cardiac fibroblasts [26]. Furthermore, Gu et al. demonstrated that GW501516 decreased the transdifferentiation of keratocytes into myofibroblasts, ECM synthesis, and corneal haze, indicating that GW501516 is capable of attenuating corneal fibrosis [54]. Contradictory data on the effect of GW501516-activated PPARδ on the myofibroblastic transition of human dermal fibroblasts were observed. The α-SMA expression was reduced in GW501516-treated human skin scleroderma fibroblasts [28], while the upregulation of α-SMA and collagen 1 was observed in human dermal fibroblasts treated with GW501516 [55]. Additionally, Kostadinova et al. described the enhancement mediated by GW501516 of various profibrotic and pro-inflammatory genes’ expressions and stimulations of hepatic stellate cell proliferation [56]. In another study, strong protection against liver fibrosis was observed in hepatocytes after KD3010 (PPARδ activation ligand) administration, whilst GW501516 had no effect on the fibrotic changes in the same model [57]. Another PPARδ agonist, HPP593, effectively reduced the renal fibrosis induced by chronic ischemia in [58]. In light of these facts, it seems that the involvement of PPARδ activation by different ligands can cause the amelioration of profibrotic changes, but the selection of an appropriate activator for a specific tissue can be difficult.

Extracellular matrix components participate in the regulation of FMT through mechanical signals that affect the actin cytoskeleton and focal adhesion sites [11]. Our previous studies indicated that TGF-β_1_-induced myofibroblastic shifts of HBFs are associated with increased levels of ECM components, such as collagens or tenascin C, as well as a highly developed actin cytoskeleton with numerous thick stress fibers accompanied by enlarged focal adhesions [23,40,59]. In this study, we showed that the administration of the PPARδ agonist GW501516 significantly attenuated collagen and tenascin C expression, as well as affected the organization of microfilament bundles, the maturation of super-mature FAs, and the expression of the FA components vinculin and talin, in TGF-β_1_-treated HBFs. Several studies indicated that PPARδ agonists inhibit the synthesis and deposition of ECM components. A significant reduction in collagens or fibronectin has been described in cultured airway smooth muscle cells, human pulmonary arterial smooth muscle cells, and mesangial cells [60,61,62]. Moreover, PPARδ agonists reduced the TGF-β_1_-induced expression of the ECM proteoglycan aggrecan in chondrocytes [63]. The disturbed maturation of FAs, as well as relatively low levels of ECM-related myofibroblast markers that affect the mechanical tension necessary for effective α-SMA-enriched stress fiber formation, was also observed in TGF-β_1_-treated HBFs in the presence of both the ligands GW501516 and GSK0660. However, the lack of a significant effect of the PPARδ antagonist GSK0660 on the FMT efficiency, myofibroblast marker expression, and FA length and composition indicates that the observed phenomenon may be, at least partially, dependent on the function of PPARδ.

Mechanistically, the regulatory role of Cx43 in the profibrotic Smad2/3 signaling during the FMT process in TGF-β_1_-treated HBFs has been previously described [39]. This study is currently the only one to show the strong inhibitory effect of PPARδ ligands administered alone or in combination on the TGF-β_1_-upregulated levels of Cx43 in HBFs from asthmatics. Attenuation of Cx43 by PPARδ ligands is associated with the disturbance of Smad2 phosphorylation rather than decreasing numbers of cells with a nuclear localization of pSmad2. Fluorometric studies revealed that the significantly upregulated pSmad2 levels in TGF-β_1_-stimulated HBFs were reduced in cells exposed to TGF-β in the presence of PPARδ ligands administered alone or in combination. The inhibition of Smad2/3 phosphorylation levels in response to GW501516 (PPARδ agonist) was observed in TGF-β-treated chondrocytes [63] or cardiac fibroblasts [29]. Thus, we suggest that the attenuation of FMT in TGF-β_1_-treated HBFs may be, at least partially, dependent on the disturbed function of the Cx43/Smad2 axis.

The enhanced phosphorylation of Smad proteins and their translocation into the cell nucleus are associated with their transcriptional activity that leads to the TGF-β-induced upregulation of myofibroblast markers (e.g., α-SMA or Cx43). The enhanced activation of Smad2 and its transcriptional function are dependent on the levels and activity of histone acetyltransferases (HATs) such as p300, which has been shown to interact with the Sry-type high mobility group box 9 (Sox9) transcription factor [46,47,64,65,66,67,68]. Through epigenetic mechanisms, p300 promotes the transcriptional activity of Smad2/3 and mediates the synthesis of myofibroblast-related proteins, which was previously described in dermal fibroblasts [69]. Moreover, Sox9’s involvement in the TGF-β-stimulated myofibroblastic transition and ECM rearrangement was observed in cardiac [70], renal [71,72], liver [73], and lung fibrosis [74]. In this study, we observed that the Smad2 phosphorylation levels upregulated by TGF-β and the increased expression of p300 and Sox9 in HBFs were strongly attenuated by GW501516 and were decreased less by the PPARδ antagonist. On the other hand, the strong inhibitory effect of GW501516 on the FMT potential, ECM-related myofibroblast markers, and FA composition, in contrast to GSK0660, suggests the involvement of PPARδ in the regulation of FMT in HBFs. However, the expression level of PPARδ did not reveal any significant differences between cells treated with TGF-β in the absence or presence of the PPARδ ligands. Therefore, we suggest that the attenuation of TGF-β-upregulated profibrotic genes and myofibroblast-related proteins in HBFs in response to GW501516 administration alone or in combination with GSK0660 may be, at least partially, the result of inhibition of the coordinated transcriptional activity of the Smad2/p300/Sox9 complex and the function of Cx43. However, we also cannot exclude the participation of PPARδ in the regulation of FMT in TGF-β-stimulated HBFs, but this hypothesis requires additional studies.

It seems that the biological effect induced by PPARδ agonists and antagonists administered simultaneously in different cell populations or whole tissues compared to that induced by PPARδ agonists administered alone may be highly dependent on the type of biological material, as well as the tested target itself (protein level, gene expression level, ROS, proliferation, scar size, speed of wound closure, etc.). Many literature data indicate that the simultaneous administration of agonists and antagonists of PPARδ shows opposite results to that of agonists alone. As described by Sng et al., the level of the LRG1 protein and transcript was increased in skin fibroblasts in response to PPARδ agonist (GW501516) administration [28]. This effect was significantly reduced in the cells treated with both a PPARδ agonist (GW501516) and antagonist (GSK0660). Similar results were observed by Gu et al. in corneal fibrosis in in vitro and in vivo models for the tested proteins: Ki67, Col1a1, Col3a1, and fibronectin [54]. The reversibility of the PPARδ agonist-induced effect by the synergistic action of a PPARδ agonist and antagonist was also described in skeletal muscle cells (C2C12 line) [35], in a mouse model of psoriasis [33], and in human umbilical vein endothelial cells (HUVEC) [34]. However, in this study, most of the observed biological targets (e.g., the tested protein levels or gene expression) in the cells exposed to the PPARδ agonist and antagonist administered simultaneously were comparable or intensified compared to those observed in the cells exposed to the agonist alone. This indicates the strong inhibitory effect of the PPARδ agonist on the tested targets. The observed effects of the PPARδ antagonist when administered alone to biological targets in TGF-β-treated HBFs were weaker and often statistically insignificant in comparison to the control TGF-β-treated cells. These observations contradict the examples cited above. This effect may be explained by the mechanism recently described by Perez Dias et al. [32]. The authors indicated the possibility of simultaneous binding of an agonist and antagonist to the PPARδ receptor in different places of the binding cassette. Changes in the conformation of PPARδ as a result of the simultaneous binding of an agonist and antagonist can promote its binding to different co-regulators and modulate the cellular response by switching the mechanisms from induction to transrepression [32]. It is possible that a similar mechanism may have been present in the experimental model used in this study, where the PPARδ agonist generated stronger cellular responses than the PPARδ antagonist through one of the mechanisms listed above. Thus, the effects of the PPARδ antagonist seem to be weaker, residing in the shadow of the agonist-induced effects. On the other hand, when an agonist and antagonist are administered simultaneously, both mechanisms may be activated in HBFs concomitantly or alternately. However, the proposed scenario is only speculation, and to precisely clarify the mechanism of action of the PPARδ agonist and antagonist (administered alone or together) in TGF-β-treated HBFs, additional studies are required.

Finally, this study is the first report showing the strong effect of a PPARδ agonist (GW501516) on the FMT potential of TGF-β_1_-stimulated HBF populations derived from asthmatic patients. It is worth highlighting that the inhibitory effect of GW501516 also affects the ECM components, FA size, and canonical TGF-β-activated profibrotic Smad2 signaling and its cooperation with the p300/Sox9 transcriptional complex. The nature of the interactions between Smad2, p300, and Sox9, and whether PPARδ is directly involved in the attenuation of TGF-β-induced FMT in HBFs remain to be elucidated. Together, the current data highlight the potential and benefits of PPARδ agonists to reduce the FMT potential, which is relevant for the clinical limitation of airway remodeling associated with asthma.

## 4. Materials and Methods

### 4.1. Cell Culture

Human bronchial fibroblasts (HBFs) isolated from bronchoscopy biopsy explants derived from asthmatic patients (*n* = 10) were established according to a previously described protocol [41]. A group of patients who qualified for this study were treated in the Department of Medicine of the Jagiellonian University Medical College in Kraków and remained in a stable clinical condition. The experimental group was characterized by the following parameters: 8 females and 2 males; age (years), 50.3 ± 17.2; BMI, 27.2 ± 3.6; FEV1%, 65.5 ± 14.6; mean duration of asthma (years), 15 ± 12.8. The study was approved by the Jagiellonian University Ethics Committee (Decision No. 1072.6120.216.2021 by M.P.). Established HBF populations were cultured in Dulbecco’s Modified Eagle Medium with high glucose (DMEM HG; Sigma-Aldrich, St. Louis, MO, USA) supplemented with 10% fetal bovine serum (FBS, Gibco, Thermo Fisher Scientific, Waltham, MA, USA) and a penicillin/streptomycin cocktail (Sigma-Aldrich, St. Louis, MO, USA). Cells were cultured in standard conditions (37 °C and 5% CO_2_). For all experiments, HBFs were used for the experiments between the 5th and 20th passages, and plated at a density of 5000 cells/cm^2^. After 24 h, the medium was replaced by serum-free DMEM HG supplemented with 0.1% bovine serum albumin (BSA, Sigma-Aldrich, St. Louis, MO, USA) in the absence or presence of the PPARδ agonist GW501516 (Sigma-Aldrich, St. Louis, MO, USA), and the PPARδ antagonist GSK0660 (Sigma-Aldrich, St. Louis, MO, USA), alone or in combination. The profibrotic response of HBFs was induced by exogenous human natural TGF-β_1_ (5 ng/mL; Corning, NY, USA). Smad activation analyses were performed in the cells exposed to TGF-β_1_ for 1 h. The gene expression, actin cytoskeleton, and focal adhesion organization were tested in the cells after 24 h of culture with the tested compounds and TGF-β_1_. Analyses of the FMT potential, Cx43 levels, cell proliferation, and viability were performed using cells cultured under the conditions described above for 4 days.

### 4.2. Proliferation and Viability Assay

HBFs were exposed to increasing concentrations of the PPARδ agonist GW501516 and the PPARδ antagonist GSK0660 alone (0–25 μM), or in combination (0–10 μM) for 4 days. Then, the cell viability was determined via a fluoresceine diacetate (FDA, Sigma-Aldrich, St. Louis, MO, USA)/ethidium bromide (EtBr, Sigma-Aldrich, St. Louis, MO, USA) assay using a Leica DMI6000B fluorescence microscope with LasX software (Leica Microsystems GmbH, Wetzlar, Germany). The results were expressed as the percentage of living cells (FDA+/EtBr-) counted under the tested conditions (5–25 μM) in relation to the control condition (0 μM). The proliferation rates of the HBF populations were determined using a crystal violet (CV, Sigma-Aldrich, St. Louis, MO, USA) assay according to a previously described protocol [40]. The absorbances of the samples were measured using a microplate reader (Multiskan FC; Thermo Fisher Scientific, Waltham, MA, USA) at λ = 540 nm. The results are presented as the mean ± standard error of the mean (SEM) of the absorbances for each tested condition.

### 4.3. Immunofluorescence Staining

Immunofluorescence studies were performed in cells growing on coverslips and fixed with 3.7% formaldehyde (Chempur, Piekary Śląskie, Poland) solution in PBS (Sigma-Aldrich) according to a previously described protocol [40]. In this study, the following primary antibodies were used: anti-α-SMA (mouse monoclonal IgG, A2547, clone 1A4, 1:400, Sigma-Aldrich, St. Louis, MO, USA), anti-vinculin (mouse monoclonal IgG, V9131, 1:200, Sigma-Aldrich, St. Louis, MO, USA), anti-connexin43 (rabbit polyclonal IgG, 1:400, Sigma-Aldrich, St. Louis, MO, USA), and anti-pSmad2^Ser465/467^ (rabbit monoclonal IgG, 18338S from Cell Signaling Technology, Danvers, MA, USA). Compatible goat anti-mouse or anti-rabbit secondary IgG antibody conjugated with Alexa Fluor 488 or Alexa Fluor 546 (1:500, Thermo Fisher Scientific, Waltham, MA, USA) was used. The cell samples were counterstained with Hoechst 33258 (1 µg/mL, Sigma-Aldrich, St. Louis, MO, USA) for DNA visualization and/or with phalloidin conjugated with Alexa Fluor 546 at the recommended concentrations for actin filament staining (Thermo Fisher Scientific, Waltham, MA, USA). The samples were mounted in fluorescent mounting medium (Dako Omnis, Agilent, Santa Clara, CA, USA) and visualized using a Leica DMI6000B microscope with LasX software (v3.7.4; Leica Microsystems GmbH, Wetzlar, Germany). All images were collected under the same parameters: time of exposition, gain, and binning. The FMT potential was determined based on the percentage of myofibroblasts with prominent α-SMA-enriched stress fibers in a population of at least 150 cells. The focal adhesion length was determined using FiJi ImageJ software (v2.1.0/1.53c; NIH, Bethesda, MD, USA). The F-actin, Cx43, and pSmad2 levels were determined fluorometrically using FiJi ImageJ software and are presented in relation to the DNA fluorescence signal.

### 4.4. Cell-Based Enzyme-Linked Immunosorbent (In-Cell ELISA) Assay

Protein content analyses were performed in methanol-fixed HBFs using an in-cell ELISA assay according to a previously described protocol [40]. Cells were incubated overnight at 4 °C with the following primary antibodies: mouse monoclonal anti-α-SMA, mouse monoclonal anti-vinculin (from Sigma-Aldrich, St. Louis, MO, USA), and rabbit monoclonal anti-talin (from Cell Signaling Technology, Danvers, MA, USA), in a dilution of 1:2000. After triple rinsing, goat anti-mouse or goat anti-rabbit secondary antibodies conjugated with horseradish peroxidase (HRP; Thermo Fisher Scientific, Waltham, MA, USA) were added and incubated at room temperature for 1 h. Then, tetramethylbenzidine (TMB, Sigma-Aldrich, St. Louis, MO, USA) was used for the induction of colorimetric reactions, which were stopped afterward using 1 N HCl/H_2_O. Absorbances were detected using a MultiskanFC microplate reader (Thermo Fisher Scientific, Waltham, MA, USA) at λ = 450 nm.

### 4.5. Western Blot Analyses

The preparation of protein lysates, protein content measurements, and Western blots were performed according to previously described protocols [40]. The membranes with the transferred proteins were incubated overnight at 4 °C with primary antibodies (mouse monoclonal IgG anti-α-SMA, rabbit polyclonal IgG anti-connexin 43, and mouse monoclonal IgG anti-β-tubulin (all Sigma-Aldrich, St. Louis, MO, USA)) diluted 1:1000 in a 1% BSA solution in Tris-buffered saline with Tween20 (TBST). After triple rinsing in TBST, the membranes were exposed to the solution of secondary antibodies (goat anti-mouse or goat anti-rabbit) conjugated with HRP (Thermo Fisher Scientific, Waltham, MA, USA) diluted 1:3000 in 2.5% skim milk/TBST. Band detection was performed using Luminata Crescendo Western HRP Substrate (Merck Millipore, Burlington, MA, USA) and a chemiluminescence imaging system ChemiDoc XRS+ (Bio-Rad, Hercules, CA, USA). Relative optical densities (RODs) were quantified with FiJi ImageJ software (v2.1.0/1.53c; NIH, Bethesda, MD, USA).

### 4.6. Real-Time PCR Analyses

The total RNA was isolated from HBFs using the RNA/miRNA GeneMATRIX UNIVERSAL purification kit (E3599-02, EURx, Gdansk, Poland) according to the manufacturer’s protocol. The reverse transcription reaction was carried out using the NG dART RT-PCR (E0801-02; EURx, Gdansk, Poland) kit and C1000 Touch Thermal Cycler (Bio-Rad, Hercules, CA, USA). For the real-time polymerase chain reaction (real-time PCR), 200 ng cDNA, the SYBR™ Green PCR Master Mix (Thermo Fisher Scientific, Waltham, MA, USA), and specifically designed primers (Table 1) were used. The PCR reactions were carried out using the 7500 Fast System (Applied Biosystems Thermo Fisher Scientific, Waltham, MA, USA). The Ct values of the tested genes refer to the Ct values of the *GAPDH* housekeeping gene and are presented as the 2^−ΔΔCt^ value ± SEM.

### 4.7. Statistics

All quantitative data are presented as the mean ± SEM. For all analyses, the normality of the distribution was estimated using the Shapiro–Wilk test. Statistical significance was tested using the nonparametric Kruskal–Wallis test with Dunn’s multiple comparisons post hoc test; * *p* < 0.05, ** *p* < 0.01, *** *p* < 0.001. All statistical analyses were performed using GraphPad Prism 5.0 software.

## Figures and Tables

**Table 1:** Primers sequences.

**Gene**	**Sequence F′**	**Sequence R′**
ACTA2	CTGTTCCAGCCATCCTTCAT	CCGTGATCTCCTTCTGCATT
COL1A1	CTTTGCATTCATCTCTCAAACTTAGTTTT	CCCCGCATGGGTCTTCA
COL1A2	TGCTGCTGGTCAACCTGGTGC	ACTTCCAGCAGGACCGGGGG
GAPDH	GAAGGTGAAGGTCGGAGT	GAAGATGGTGATGGGATTTC
TLN	CCCTGATGTGCGGCTTCG	TGTCCTGTCAACTGCTGCTTC
TNC	GGTCCACACCTGGGCATTT	TTGCTGAATCAAACAACAAAACAGA
GJA1	AGGAGTTCAATCACTTGGCG	GAGTTTGCCTAAGGCGCTC
PPARD	GGGCATGTCACACAACGCTAT	GCATTGTAGATGTGCTTGGAGAA
P300	ACTTCTAATGGCCCTCTACCTGA	GTGCTGAAGAGGAGGGGTTT
SOX9	CAAGAAGGACCACCCGGATT	AAGATGGCGTTGGGGGAGAT

## Data Availability

All data supporting reported results are available upon request from the corresponding author.
